# Design, Modeling, and Visual Learning-Based Control of Soft Robotic Fish Driven by Super-Coiled Polymers

**DOI:** 10.3389/frobt.2021.809427

**Published:** 2022-03-04

**Authors:** Sunil Kumar Rajendran, Feitian Zhang

**Affiliations:** ^1^ Department of Electrical and Computer Engineering, Volgenau School of Engineering, George Mason University, Fairfax, VA, United States; ^2^ Department of Advanced Manufacturing and Robotics, Peking University, Beijing, China

**Keywords:** underwater robots, soft robotics, fish swimming, bio-inspired robotics, artificial muscle, deep reinforcement learning, convolutional neural network (CNN)

## Abstract

A rapidly growing field of aquatic bio-inspired soft robotics takes advantage of the underwater animals’ bio-mechanisms, where its applications are foreseen in a vast domain such as underwater exploration, environmental monitoring, search and rescue, oil-spill detection, etc. Improved maneuverability and locomotion of such robots call for designs with higher level of biomimicry, reduced order of complex modeling due to continuum elastic dynamics, and challenging robust nonlinear controllers. This paper presents a novel design of a soft robotic fish actively actuated by a newly developed kind of artificial muscles—super-coiled polymers (SCP) and passively propelled by a caudal fin. Besides SCP exhibiting several advantages in terms of flexibility, cost and fabrication duration, this design benefits from the SCP’s significantly quicker recovery due to water-based cooling. The soft robotic fish is approximated as a 3-link representation and mathematically modeled from its geometric and dynamic perspectives to constitute the combined system dynamics of the SCP actuators and hydrodynamics of the fish, thus realizing two-dimensional fish-swimming motion. The nonlinear dynamic model of the SCP driven soft robotic fish, ignoring uncertainties and unmodeled dynamics, necessitates the development of robust/intelligent control which serves as the motivation to not only mimic the bio-mechanisms, but also mimic the cognitive abilities of a real fish. Therefore, a learning-based control design is proposed to meet the yaw control objective and study its performance in path following *via* various swimming patterns. The proposed learning-based control design employs the use of deep-deterministic policy gradient (DDPG) reinforcement learning algorithm to train the agent. To overcome the limitations of sensing the soft robotic fish’s states by designing complex embedded sensors, overhead image-based observations are generated and input to convolutional neural networks (CNNs) to deduce the curvature dynamics of the soft robot. A linear quadratic regulator (LQR) based multi-objective reward is proposed to reinforce the learning feedback of the agent during training. The DDPG-based control design is simulated and the corresponding results are presented.

## 1 Introduction

The nascent field of bio-inspired robotics has gained a huge popularity over the past 2 decades with numerous designs and developments contributed to the community (Pfeifer et al., 2007; Kim et al., 2013; Shi et al., 2015; Laschi et al., 2016; Christianson et al., 2019; Olsen and Kim, 2019), envisioning their applications in domains such as environmental monitoring, deep-sea exploration, search and rescue, and disaster response (Morgansen et al., 2007; Zheng Chen et al., 2010; Marchese et al., 2014; Phamduy et al., 2015). Taking advantage of natural biological structures, functions, and motions of aquatic animals aids us in creating underwater robots which are energy and locomotion efficient, and possess agile maneuverability, for a diverse range of purposes. Our research focuses on developing a biomimetic underwater soft robotic fish that can self-learn its locomotion to achieve different goals such as regulating its angle of orientation and adapting to variable swimming speeds (Rajendran and Zhang, 2018), which eventually serve as decomposed control tasks for high-level control objectives such as traversing along a planned trajectory and studying fish swarming behavior like schooling and shoaling.

The biological fish that employ body/caudal fin for propulsion typically adopt one of the following swimming styles, namely carangiform, sub-carangiform, anguilliform, and thunniform (Videler, 1993). Most of the traditional robotic fish prototypes designed in the past, comprise of two or more serially connected structures (Wen et al., 2012; Zhong et al., 2017), whose coordinated discrete movements result in undulations mimicking one of these swimming styles. The body of these robots are structurally constructed using rigid materials such as plastic, metal and glass-fiber (Raj and Thakur, 2016), which consequently increases the rigidity and mass of the robot. To overcome this limitation, over the past demi-decade, researchers have been exploring the usage of soft materials (Lauder et al., 2011) such as silicone rubber/elastomer (Katzschmann et al., 2018), silicone prepolymer (Aubin et al., 2019) and silk hydrogel (Donatelli et al., 2018) to construct the body of the fish robot (Olsen and Kim, 2019). The adoption of such soft materials in the construction of the robotic fish greatly contributes towards mimicking the flexibility of the biological fish body, thus generating a continuous deformation and streamlined displacement of water.

Traditional actuators such as electrical motors and pneumatic/hydraulic cylinders which are employed to realize fish undulations in the aforementioned multi-link robotic fish prototypes, although offer a high output force/torque, are generally heavy and quite rigid, thus making fish robots less flexible. Hence, the use of soft actuators such as artificial muscles like pneumatic artificial muscles (PAM), ionic polymer-metal composites (IPMC) (Chen, 2017; Olsen and Kim, 2019), dielectric elastomer actuators (Christianson et al., 2019), and super-coiled polymers (SCP) (Yip and Niemeyer, 2017; Rajendran and Zhang, 2018; Simeonov et al., 2018) is on the rise. Not only are artificial muscles slender, but also strong, flexible, lightweight, and analogously compliant to biological muscles. This offers appealing advantages to fish robots in terms of flexibility, maneuverability, propulsive energy efficiency and the ability to precisely mimic the biological fish from its anatomical perspective.

Over the past 3 decades, researchers from a wide field of disciplines have performed numerous visual experiments and numerical analysis to study and model the various swimming styles in different species of fish (Triantafyllou et al., 2000; Lauder, 2015; Webb and Gerstner, 2021). Most of the traditional models follow Lighthill’s elongated-body theory describing fish locomotion as traveling waves (Lighthill, 1971), or employ a mathematical dynamic model derived *via* system identification. As contemporary research focuses on mimicking the physical and biological structure and function of aquatic animals using soft materials, the necessity of arriving at a precise dynamic model for motion prediction and controller design is also simultaneously increasing. Nevertheless, this is becoming correspondingly difficult due to the continuum dynamics and high dimensionality involved in soft robots.

While different classical and modern control techniques have been analytically researched and experimentally developed, the nonlinearity of contemporary soft robots keeps rising continuously. As several robotic fish prototypes adopt various closed-loop control techniques such as PID control (Yu et al., 2004; Berlinger et al., 2021), PI control (Zhang et al., 2015a), central pattern generator control (Jeong et al., 2011), pre-trained neural networks (Thuruthel et al., 2019), robust control (Zhang et al., 2015b), to improve the performance of locomotion, others employ open-loop control techniques whereby a predefined swimming profile is generated to perform a coded set of actions (lookup table) which is predominantly used in cases of complex or highly nonlinear robotic fish dynamic models (Yu and Wang, 2005; Korkmaz et al., 2012). However, in order to address the problems of high nonlinearity and intrinsically infinite system dimension, researchers are looking into various present-day techniques in artificial intelligence (Rajendran and Zhang, 2018; Bhagat et al., 2019; Thuruthel et al., 2019), more specifically behavior-based or adaptive machine learning-based control.

Our previous work investigated the performance of SCP actuators while submerged in water and the compatibility of using SCP in a simple robotic fish model (Rajendran and Zhang, 2017). SCP, a recently developed artificial muscle actuator, is lightweight, flexible, strong with a high power-to-weight ratio and fabricated with silver-plated nylon threads (Yip and Niemeyer, 2017). Our study also showed through simulation that speed control of a one-dimensional robotic fish was successfully done with SCP actuators using reinforcement learning (Rajendran and Zhang, 2018; Sutton and Barto, 2018). Nevertheless, besides employing a sparsely discretized state space in the dynamics, our previous model is dimensionally limited which is too simplified to mimic the biological fish and study the swimming motion. This enforced the use of a lookup table which comprised of all the state-action combinations. However, since physical robots comprise of continuous action and state spaces, the use of Q-learning algorithm (Watkins and Dayan, 1992) in such a continuous environment would require an enormous lookup table, as a result, drastically increasing the number of computations.

In this paper, we propose a novel approach in designing a soft robotic fish using antagonistically arranged SCP artificial muscle actuators. The soft robotic fish is modeled geometrically as a three-link model combined with the antagonistic configuration of the SCP muscles, and modeled dynamically by incorporating the SCP actuator dynamics (Rajendran and Zhang, 2017; Yip and Niemeyer, 2017) with the hydrodynamic forces (Wang et al., 2015) to describe its two-dimensional swimming motion. To overcome the predicament of having a highly nonlinear and multi-dimensional control system, in addition to consideration of control computation times, this paper proposes a learning-based controller design approach for the dynamically modeled soft robotic fish using an improved, continuous reinforcement learning method, namely deep deterministic policy gradient (DDPG) algorithm (Lillicrap et al., 2015), which adopts an actor network to perform an action given a state, and a critic network to criticize the chosen action. To exemplify the use of DDPG in the dynamic model, this paper investigates the closed-loop control of the swimming orientation and path following of the soft robotic fish on a 2D plane.

This paper is organized as follows. Section 2 gives a brief overview on the experimental performance of SCP muscles when submerged in water. Section 3 presents the design of a three link soft robotic fish and its two-dimensional dynamic model. Section 4 illustrates and elucidates the geometric and dynamic model of the robotic fish. Section 5 proposes the deep-deterministic policy gradient learning based control design for the soft robotic fish to self-learn its swimming profiles to regulate the orientation and achieve path following by the fish. Simulation results are presented to validate the proposed controller design in Section 6. Finally, conclusion remarks are provided in Section 7.

## 2 Preliminary Background

**FIGURE 1:**
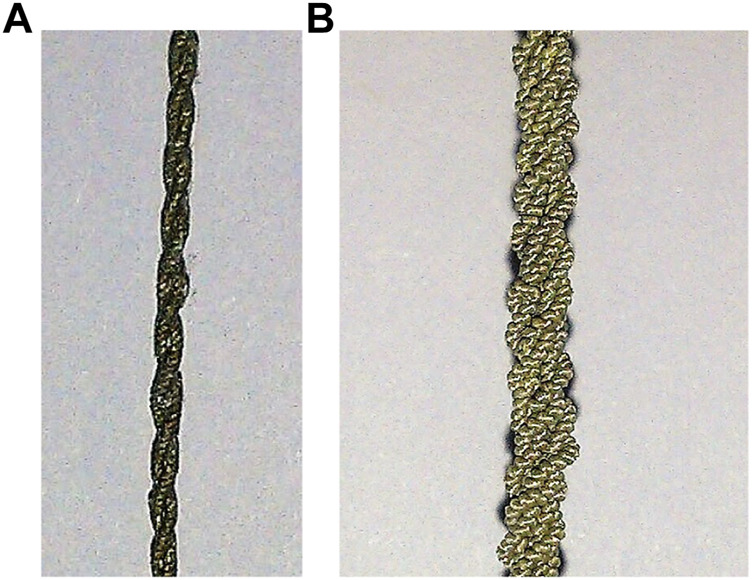
SCP artificial muscles (Rajendran and Zhang, 2017). **(A)** One 2-ply SCP muscle coated with silicone and acrylic caulk; **(B)** three 2-ply SCP muscles twined together.

Our previous work presented a two-link flapping prototype driven by an SCP muscle actuator and investigated its performance by submerging and testing the entire two-link prototype in ordinary non-deionized non-conductive tap water at room temperature (Rajendran and Zhang, 2017). As a proof of concept of the SCP actuation, we conducted the experiment using one 2-ply muscle as shown in Figure 1A, which was attached to one side of the two-links connecting both the ends spaced at 2.5 cm away from the links. Initially, only a little deformation (less than 0.5%) was observed in the SCP actuators when immersed in water. We conjecture that this comes from the fast heat dissipation in water, which eventually causes the muscle to hardly contract. To overcome this problem the muscle was coated with silicone conformal spray along with a layer of siliconized acrylic caulk as shown in Figure 1B and also a higher voltage (2 V per centimeter of the muscle) for excitation was applied. This resulted in a deformation of around 1%, eventually causing the flap angle to change by 16 degrees approximately. Moreover, the time taken for the flap to return to its original position was around 2 s on average, which is five times faster than when tested in air. From the results, it was evident that the recovery speed of the SCP actuator was significantly improved when tested in water. However, the maximum attainable flap angle became smaller in water. Also, a higher voltage had to be applied to the SCP actuator thus consuming more power. Having made these inferences, it comes to a design trade-off between actuation/recovery speed and energy consumption when using enhanced SCP actuators for underwater robots like robotic fish. With the proposed antagonistic design and muscle contraction in alternating directions, fish-like swimming is achievable with the SCP actuators.

Following this, aiming towards a phased approach at developing reinforcement learning-based control for the soft robotic fish, a foundational Q-learning (Watkins and Dayan, 1992) based controller was designed and simulated to control the speed of a three-link robotic fish which consisted of discretized state and action spaces (Rajendran and Zhang, 2018). The robotic fish was restricted to one-dimensional locomotion and the agent was trained until the Frobenius norm between the current and previous Q-tables was minimized to a threshold. We observed from the simulation results that the robotic fish followed the learned swimming profile and regulated the speed to the reference value with a very small speed control error. Eventually, the averaged acceleration became zero, thus maintaining a quasi-steady-state forward swimming velocity. Another interesting observation was that the agent forcefully went to its resting state, i.e., all actuators at rest, in order to lower the speed when it exceeded the desired velocity. Likewise, with different desired velocities, we found a difference in the flapping frequency and amplitude. Considering the coarse scale of discretization, we consider the learning based speed control design succeeded in the simulation example, thus promising a scope to design advanced learning-based controllers for continuous action and state spaced robots.

## 3 Design of a 3-Link Soft Robotic Fish

**FIGURE 2:**
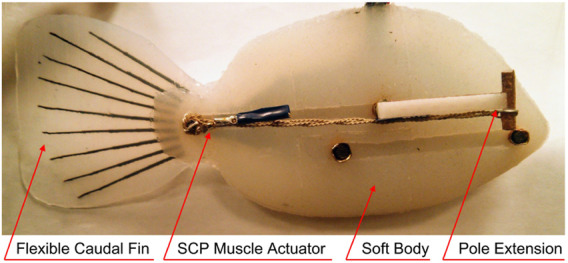
Soft robotic fish with passive caudal fin, bundled SCP actuator and pole extensions attached.

**FIGURE 3:**
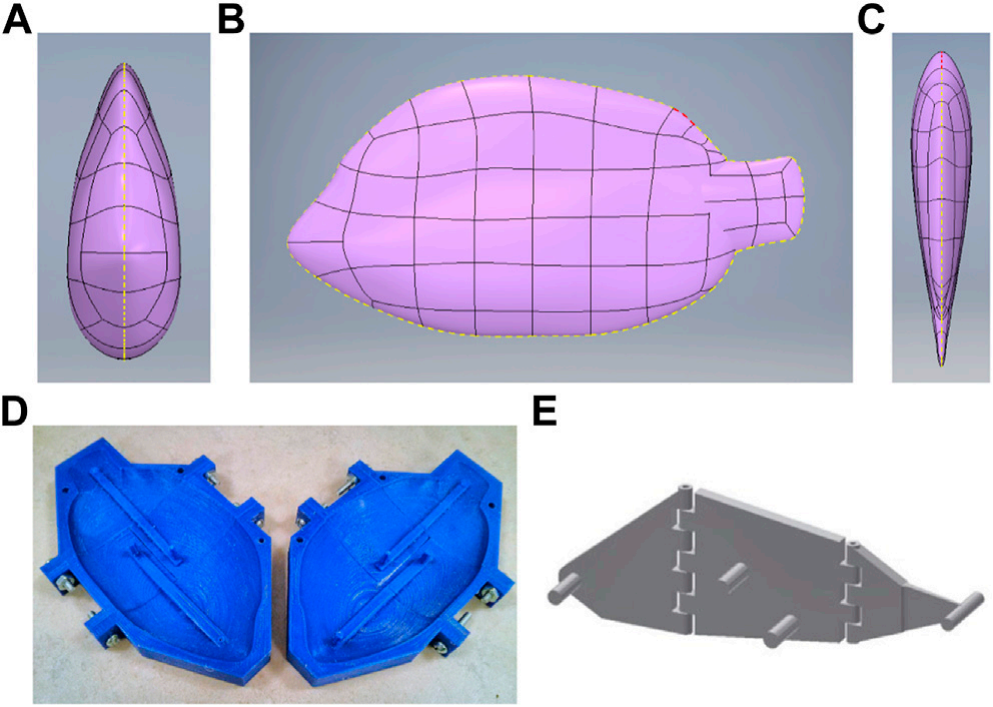
Soft robotic fish design components. **(A–C)** Illustration of the robotic fish CAD design, from left to right: front, side and top views (Rajendran and Zhang, 2018); **(D)** 3D-printed fish molds (Rajendran and Zhang, 2018); **(E)** 3-link hinged attachment.

The design of our soft robotic fish as shown in Figure 2, is inspired by the natural and biological structure of Tilapia cichlid fish species, which is specifically chosen to moderate the amount of volumetric material in the construction of the soft robotic fish body, and to build a lighter robot for greater maneuverability. The entire 3D model of the fish is designed using freeform modeling in AutoDesk Inventor, by tracing the front, side and top views of the cichlid fish as shown in Figures 3A–C, to maintain the shape of a streamlined body. Two symmetric molds are designed based on the generated CAD fish model and then 3D printed using PLA filament as shown in Figure 3D. These molds are then casted with Ecoflex 00–20 silicone rubber by Smooth-On with a curation period of 4 h.

Once the silicone rubber bodies are cured, three links which form the skeletal bone of the fish to provide rigidity to the robot’s soft body in the process of actuation, are designed and 3D printed. The three links are attached in series together using the hinges on the links as shown in Figure 3E and by inserting straightened steel paper clips to provide a medium of pivoting. To form the electrical connections, steel crimps and copper tapes are attached around the poles on both sides of the links. The poles on the first and third links are connected together to form the common ground terminal. Long flexible wires are connected to the rest of the four poles on the second link, and one wire to the ground terminal, resulting in five wires that exit the robot.

To increase the propulsion efficiency of the robot, a truncated flat type passive caudal fin is attached close to link three using a flexible silicone rubber adhesive. This fin is casted on a 3D designed and printed shallow mold, using the same silicone rubber material. Within 12 min of the material being casted, thinly 3D printed semi-flexible rods which mimic the fin rays in a caudal fin are placed on a growing fashion in the casted mold, so that the fin rays are submerged, thus forming a semi-flexible caudal fin once cured. Two pole extensions are attached on the newer version of our soft robotic fish in order to provide more room for the bundled SCP actuator, consequently exhibiting more deformation in the actuator resulting in higher deflection of the tail. The pole extensions also have the ability to house multiple actuators in parallel.

## 4 3-Link Robotic Fish Model

The soft robotic fish is modeled from its geometrical and dynamical perspectives. In this paper, the soft robotic fish is constrained to a planar swimming motion, thus fixating its altitude.

### 4.1 Geometric Model

**FIGURE 4:**
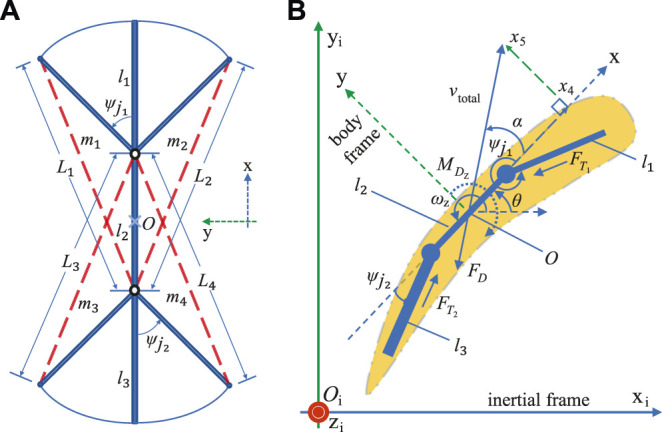
Robotic fish modeling. **(A)** Geometric model schematic; **(B)** dynamic model schematic.

The geometry of the 3-link fish robot with the artificial muscle actuators attached, is illustrated in Figure 4A, is defined with respect to the soft robotic fish’s body or local reference frame $F_{\text{b}}$ with 2D Cartesian coordinates given by (x, y). The fish robot is modeled as three serially connected rigid links *l*_1_, *l*_2_ and *l*_3_, which correspond to the head, body and tail links respectively, thus forming joints *j*_1_ and *j*_2_. Link *l*_2_ is orthogonal to the y axis and fixed to the x axis in the body frame with its center defined as the origin *O* of body frame. Four SCP muscle actuators *m*_1_, *m*_2_, *m*_3_, and *m*_4_, whose current lengths are given by *L*_1_, *L*_2_, *L*_3_, and *L*_4_, connect the ends of the subsequent pairs of links (*l*_1_, *l*_2_) and (*l*_2_, *l*_3_) on either side thus forming two agnostic-antagonistic muscle pairs, as illustrated in Figure 4A. With the lengths of the three links denoted as |*l*_1_|, |*l*_2_|, and |*l*_3_|, the length of a muscle *m*_*i*_ is expressed as

$$L_{i}=d_{i}{\left(\left|l_{1}|+|l_{2}\right|\right)}^{[[i\in \left\{1,2\right\}]]}{\left(\left|l_{2}|+|l_{3}\right|\right)}^{\left[[i\in \left\{3,4\right\}]\right]},$$ (1)

where *d*_*i*_ is the deformation ratio between the current and original resting length of a muscle *m*_*i*_ satisfying *i* ∈ (1, 2, 3, 4), and [[ (⋅) ]] denotes the Iverson bracket such that [[ (*condition*) ]] = 1 when the *condition* is true and equal to 0 otherwise (Knuth, 1992). The coordinated actuation of these SCP muscles causes deformation with respect to their lengths, consequently, causing flapping movements of the links *l*_1_ and/or *l*_3_ with respect to link *l*_2_. The angles formed due to the rotations of links *l*_1_ and *l*_3_ around joints *j*_1_ and *j*_2_ are denoted by the flap or deflection angles $\psi_{j_{1}}$ and $\psi_{j_{2}}$ , following Fleming’s right hand rule. The geometric model defining these two angles can be summarized by the expressions

$$\psi_{j_{1}}={\left(-1\right)}^{\delta_{i2}}\cos^{-1}{\left(\frac{L_{i}^{2}-|l_{1}{|}^{2}-|l_{2}{|}^{2}}{2|l_{1}\|l_{2}|}\right)}^{\left[[i\in \left\{1,2\right\}]\right]},$$ (2)

$$\psi_{j_{2}}={\left(-1\right)}^{\delta_{i3}}\cos^{-1}{\left(\frac{L_{i}^{2}-|l_{2}{|}^{2}-|l_{3}{|}^{2}}{2|l_{2}\|l_{3}|}\right)}^{\left[[i\in \left\{3,4\right\}]\right]},$$ (3)

where *δ*_*i*2_ and *δ*_*i*3_ are Kronecker delta functions, and *i* represents the current muscle which is activated. From past research conducted by fish biologists and roboticists, a maximum oscillatory amplitude by a flap angle of 25° is adequate (Zhong et al., 2017) to achieve a considerable swimming speed of the robotic fish, and is easily achieved in the aforementioned geometric model with a deformation of an SCP muscle reaching as low as 2.5*%* or *d*_*i*_ = 0.025 (Rajendran and Zhang, 2017; Rajendran and Zhang, 2018), provided that the muscles are placed close to the links unlike the experimental prototype described in Section 2.

### 4.2 Dynamic Model

The schematic of the soft robotic fish along with relevant reference frames and variables that describe the motion of the robot is illustrated in Figure 4B. The inertial or stationary frame of reference is denoted by $F_{\text{i}}$ which comprises of 3D Cartesian coordinates (x_i_, y_i_, z_i_) and origin *O*_i_, and represents all of the global positions and orientations of the fish. The origin of the body frame *O* also corresponds to the center of mass of the robotic fish. The dynamic model of the soft robotic fish employed in this paper encompasses the dynamics of the SCP actuator, the geometry of the 3-link fish model, and the hydrodynamic forces which include the drag and thrust with respect to the planar dynamics of the soft robotic fish.

The entire dynamics of the soft robotic fish driven by artificial muscles is modeled using two subsystems. The first subsystem comprises of the thermo-electrical and thermo-mechanical dynamics of the SCP muscle actuators which takes in the actuating voltage potentials and outputs the deformations in the muscles’ lengths (Yip and Niemeyer, 2017). The system input vector is given by $u={\left[u_{1},u_{2}\right]}^{\text{T}}={\left[-V_{1}+V_{2},-V_{3}+V_{4}\right]}^{\text{T}}$ , where *V*_*i*_ represents the actuating voltage potential applied to the muscle *m*_*i*_ where *i* ∈ (1, 2, 3, 4). The antagonistic arrangement of the muscles restricts actuation to only one or none of the muscles in the pairs (*m*_1_, *m*_2_) and/or (*m*_3_, *m*_4_) at a time, consequently holding the expression *V*_1_ *V*_2_ = *V*_3_ *V*_4_ = 0 true at all times. The system dynamics of the SCP actuator derived from (Yip and Niemeyer, 2017; Rajendran and Zhang, 2018) are incorporated in this model to suit the antagonistic configuration of the actuators. The dynamics mainly include the change in muscle length Δ*L*_*i*_, rate of change in muscle length $\dot{\Delta L_{i}}$ and change in temperature Δ*T*_*i*_ with respect to the ambient temperature *T*_0_ of the actuator *m*_*i*_ where *i* ∈ (1, 2, 3, 4). Due to the antagonistic configuration we consider Δ*L*_1_ = −Δ*L*_2_ and Δ*L*_3_ = −Δ*L*_4_. The states of the SCP actuator subsystem can be collectively put as $x_{m_{i}}={\left[\Delta L_{i},\dot{\Delta L_{i}},\Delta T_{i}\right]}^{\text{T}}={\left[x_{m_{i,1}},x_{m_{i,2}},x_{m_{i,3}}\right]}^{\text{T}}$ where *i* ∈ (1, 2, 3, 4). The complete dynamic model of the SCP actuator subsystem is then given by

$${\dot{x}}_{m_{i,1}}=x_{m_{i,2}},$$ (4)

$$\begin{array}{l} {\dot{x}}_{m_{i,2}}=\frac{{\left(-1\right)}^{\left[\;\left[i\in \left\{1,3\right\}\right]\;\right]}}{M_{m}}{\left(F_{m_{2}}-F_{m_{1}}\right)}^{\left[[i\in \left\{1,2\right\}]\right]}\\ {\left(F_{m_{4}}-F_{m_{3}}\right)}^{\left[\;\left[i\in \left\{3,4\right\}\right]\;\right]}, \end{array}$$ (5)

$${\dot{x}}_{m_{i,3}}=\frac{u_{1}^{2\left[\;\left[i\in \left\{1,2\right\}\right]\;\right]}u_{2}^{2\left[\;\left[i\in \left\{3,4\right\}\right]\;\right]}-\lambda R_{m}x_{m_{i,3}}}{C_{\text{th}}R_{m}},$$ (6)

where *M*_*m*_ is the mass of the SCP muscle actuator, *λ* is the absolute thermal conductivity, *R*_*m*_ is the electrical resistance of the actuator, *C*_th_ is the coefficient of thermal mass, $F_{m_{i}}$ is the force generated by the muscle *m*_*i*_ where *i* ∈ (1, 2, 3, 4) and is given by

$$F_{m_{i}}=c_{m}x_{m_{3}}-k_{m}x_{m_{1}}-b_{m}x_{m_{2}},$$ (7)

where *b*_*m*_ is the damping coefficient, *c*_*m*_ is the thermal constant and *k*_*m*_ is the mean stiffness constant of the SCP actuator.

The deformed lengths of the muscles are used to derive the soft robotic fish’s profile or discretized curvature in its body frame using the 3-link geometric model as equated in Eqs. 1–3. Consequently, the joint angles establish the input to the second subsystem which comprises of the planar positional dynamics and hydrodynamics of the robotic fish. The states of the second subsystem are collectively given by the vector $x={\left[x_{\text{i}},y_{\text{i}},\theta ,v_{\text{x}},v_{\text{y}},\omega_{\text{z}}\right]}^{\text{T}}={\left[x_{1},x_{2},\ldots x_{6}\right]}^{\text{T}}$ , where x_i_, y_i_, and *θ* represent the pose (2D Cartesian coordinate position and orientation) of the robot respective to its inertial frame $F_{\text{i}}$ , and $v_{\text{x}}$ , $v_{\text{y}}$ , and *ω*_z_ represent the surge, sway and angular velocities of the robot respective to its body frame $F_{\text{b}}$ . The angular velocity of fish is also termed as swinging motion (Farideddin Masoomi et al., 2015). The output vector of the entire soft robotic fish system is given by $y={\left[\theta ,\omega_{\text{z}},\alpha ,v_{\text{total}}\right]}^{\text{T}}$ , which is primarily considered in the design of the learning-based controller to implement various control objectives. In the aforementioned system output vector, the angle of attack of the robotic fish is expressed as *α* = tan^−1^ (*x*_5_/*x*_4_), and $v_{\text{total}}=\sqrt{x_{4}^{2}+x_{5}^{2}}$ is the swimming velocity of the robotic fish. The kinematic and dynamic model of the soft robotic fish is then equated by

$$\dot{x}=\left[\begin{array}{c} x_{4}\cos x_{3}-x_{5}\sin x_{3}\\ x_{4}\sin x_{3}+x_{5}\cos x_{3}\\ x_{6}\\ \frac{\left(M_{f}+M_{\text{y}}\right)x_{5}x_{6}+F_{\text{x}}}{M_{f}+M_{\text{x}}}\\ \frac{-\left(M_{f}+M_{\text{x}}\right)x_{4}x_{6}+F_{\text{y}}}{M_{f}+M_{\text{y}}}\\ \frac{\tau_{\text{z}}}{J_{\text{z}}} \end{array}\right]$$ (8)

where *M*_*f*_ is the mass of the robotic fish, *M*_x_ and *M*_y_ are the added masses along the x and y directions respectively, *J*_z_ is the mass moment of inertia of the robotic fish about the z axis, *F*_x_ and *F*_y_ are the forces acting along the x and y directions in the body frame, and *τ*_z_ is the moment or torque about the z axis. These forces and moment are expressed as

$$\begin{array}{c} F_{\text{x}}=-F_{T_{1}}\cos\psi_{j_{1}}+F_{T_{2}}\cos\psi_{j_{2}}-F_{D}\cos\alpha \\ \;+F_{L}\sin\alpha , \end{array}$$ (9)

$$\begin{array}{c} F_{\text{y}}=F_{T_{1}}\sin\psi_{j_{1}}+F_{T_{2}}\sin\psi_{j_{2}}-F_{D}\sin\alpha \\ \;-F_{L}\cos\alpha , \end{array}$$ (10)

$$\tau_{\text{z}}=M_{D_{z}}+F_{T_{1}}K_{M_{1}}\sin\psi_{j_{1}}+F_{T_{2}}K_{M_{2}}\sin\psi_{j_{2}},$$ (11)

where $F_{T_{1}}$ and $F_{T_{2}}$ are the hydrodynamic thrust forces exerted due to rotations of the links *l*_1_ and *l*_3_ around joints *j*_1_ and *j*_2_ respectively. *F*_*D*_ is the hydrodynamic drag force acting on the opposite direction of the robot, and *F*_*L*_ is the lift force acting orthogonal to the robot which contribute predominantly to the forward motion of the robot. $M_{D_{\text{z}}}$ is the damping factor of the moment and $K_{M_{1}}$ and $K_{M_{2}}$ are the moment coefficients of joint *j*_1_ and *j*_2_ respectively. The hydrodynamic forces of the robotic fish follow (Wang et al., 2015) and are determined from

$$F_{D}=\left(K_{D}+K_{D_{\alpha }}\alpha^{2}\right)v_{\text{total}}^{2},$$ (12)

$$F_{L}=K_{L}\alpha v_{\text{total}}^{2},$$ (13)

$$M_{D_{\text{z}}}=-K_{M}\omega_{\text{z}}^{2}\text{sgn}\left(\omega_{\text{z}}\right),$$ (14)

$$F_{T_{1}}=K_{j_{1}}|l_{1}|{\dot{\psi_{j_{1}}}}^{2},$$ (15)

$$F_{T_{2}}=K_{j_{2}}|l_{3}|{\dot{\psi_{j_{2}}}}^{2}.$$ (16)

Here, *K*_*D*_ is the drag coefficient of the soft robotic fish body, $K_{D_{\alpha }}$ is the drag coefficient pertaining to the swimming direction respective to the body frame, *K*_*L*_ is the lift coefficient, *K*_*M*_ is the damping coefficient with respect to the rotational velocity *ω*_z_ in the body frame of the robot, $K_{j_{1}}$ and $K_{j_{2}}$ are the thrust force coefficients pertaining to joints *j*_1_ and *j*_2_, and their corresponding flapping angular velocities $\dot{\psi_{j_{1}}}$ and $\dot{\psi_{j_{2}}}$ are obtained by taking the time derivatives of the head and tail flap angles $\psi_{j_{1}}$ and $\psi_{j_{2}}$ that are expressed in Eqs 2, 3 respectively, thus giving

$$\dot{\psi_{j_{1}}}={\left(-1\right)}^{\delta_{i1}}{\left(\frac{2L_{i}x_{m_{2}}}{2|l_{1}\|l_{2}|\sqrt{1-\cos^{2}\left(\psi_{j_{1}}\right)}}\right)}^{\left[[i\in \left\{1,2\right\}]\right]},$$ (17)

$$\dot{\psi_{j_{2}}}={\left(-1\right)}^{\delta_{i4}}{\left(\frac{2L_{i}x_{m_{2}}}{2|l_{2}\|l_{3}|\sqrt{1-\cos^{2}\left(\psi_{j_{2}}\right)}}\right)}^{\left[[i\in \left\{3,4\right\}]\right]}.$$ (18)

The aforementioned soft robotic fish dynamics is approximated as a simplified three-link model, which ignores the fluid structure interactions, however, considers the hydrodynamic forces of robotic fish per se in its dynamic model. The fish prototype presents its own limitation such as bounded tail-flapping range due to the geometric constraints involving the SCPs, thus restricting the range of undulations too. Additionally, the actuation frequency of the soft robotic fish is implicitly restricted by taking the SCP dynamics into consideration, whereby the SCP’s time constant approximates to 0.8 s when submerged in water (Rajendran and Zhang, 2017), thus bounding the upper actuation frequency to ≤1.25 Hz.

## 5 Motion Planning of Soft Robotic Fish Using Learning-Based Control

This section aims at designing a learning-based controller to meet various motion planning control objectives of the soft robotic fish which includes 1) regulating the yaw angle *θ* and 2) path following *via* tracking given waypoints. Nevertheless, the consolidated dynamics of the various subsystems constituting the soft robotic fish model as given in Eqs 4–18, is fairly complex and nonlinear, exhibits hysteresis, and uncertainties usually in dynamics of the actual systems, thus necessitating a robust nonlinear controller. To alleviate the challenges which mostly arise in designing a traditional nonlinear controller, this paper combines a contemporary reinforcement learning algorithm from the field of artificial intelligence and a customized framework to design a learning-based controller. In contrast to the simple Q-learning based approach employed in our previous work (Rajendran and Zhang, 2018), this paper adopts a much more sophisticated and efficient deep reinforcement learning algorithm called deep-deterministic policy gradient algorithm (DDPG), which is compatible with continuous action and state spaces (Lillicrap et al., 2015). The following subsections describe the architecture of the learning framework consolidating the aforementioned soft robotic fish model with the learning environment, and gives an overview of DDPG reinforcement learning algorithm, the deployed reward function and hyper-parameters.

### 5.1 Learning Framework and Architecture

#### 5.1.1 Agent and Environment

The inherent cognitive realization of the soft robotic fish is characterized as a learning agent that takes in the current system state ***s*** obtained from feedback of the robot and outputs the best possible action ***a***. The learning agent primarily constitutes of an actor deep neural network (DNN), which is iteratively trained using the DDPG learning algorithm. An action performed by the agent at any given time instant, comprises of the voltage potential *V*_*i*_ applied to the SCP actuators *m*_*i*_ where *i* ∈ (1, 2, 3, 4). The action vector follows the system input vector as defined before in the dynamic model in Section 3, which is collectively put as $a={\left[u_{1},u_{2}\left.\right||u_{1}|\leq V_{\text{max}},|u_{2}|\leq V_{\text{max}}\right]}^{\text{T}}\in R^{2}$ , and is bounded by a maximum voltage potential *V*_max_ that is applicable to an actuator such that *V*_*i*_ ∈ (0, *V*_max_). The agent’s actions and states are defined in the continuous action and state spaces denoted by A and S respectively. The agent’s state is defined as ***s*** = *f* (***ψ***, ***x***, ***y*** ^∗^) which is a function of the soft robot’s curvature dynamics (joint angles and flapping angular velocities) given by $\psi =\left[\psi_{j_{1}},\dot{\psi_{j_{1}}},\psi_{j_{2}},\dot{\psi_{j_{2}}}\right]$ , dynamic system state vector ***x*** that corresponds to the soft robotic fish and the system output reference vector ***y*** ^∗^. The significance of including the flap angles and angular velocities in the agent’s state vector, lies in the necessity to provide the agent with the knowledge of the robot’s 3-link discretized curvature or profile in its body frame, and which is also proportionally related to the SCP muscle dynamics. The agent’s environment encompasses the system dynamics and state progression of the soft robotic fish which consequently outputs an evaluation of the newly transitioned state in the form of reinforcements.

#### 5.1.2 Image-Based Observations

Foreseeing the experimental validation on the physical soft robotic fish, most of the states in ***s***, necessary for the agent to envision the robot’s pose, can be obtained through feedback *via* electronic sensing by embedding various position sensors such as inertial measurement unit, accelerometer, and/or gyroscope. Obtaining the curvature of the soft robotic fish is equally indispensable for the agent to envision the robot’s profile, however, employing the use of flex sensors or distributed sensing elements in/around the soft body has its own limitations. While flex sensors require a complex arrangement/construction to maximize the frictional and spatial contact between the sensor strip and the soft body, use of distributed sensing elements such as pressure sensors not only limits to a finite set of discretized measurements of the soft body profile in contrast to its continuum curvature, but also requires an optimal position of sensor placement.

**FIGURE 5:**
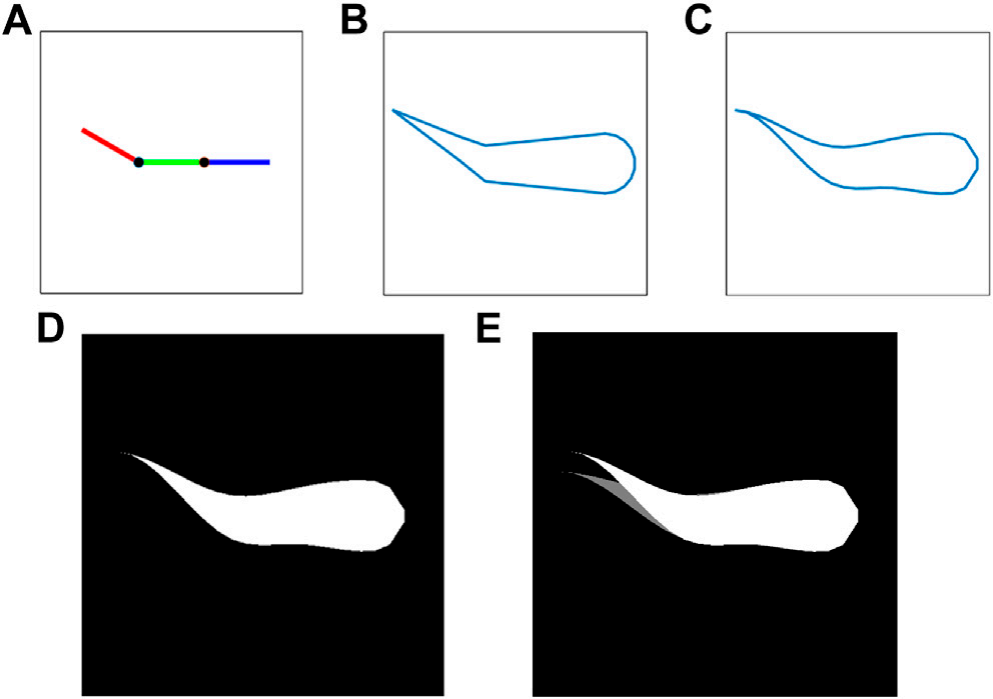
Sequential approach towards generating an image-based observation ***z***_*p*,*q*_(*t*) of a sample soft robotic fish profile with $\psi_{j_{1}}=0{}^{\circ}$ and $\psi_{j_{2}}=-30{}^{\circ}$ at time *t*. **(A)** Geometric plot of 3-link robotic fish; **(B)** generating a perimetric offset around the three links; **(C)** cubic spline interpolation of the perimetric offset; **(D)** generated Region of Interest by masking the interpolated closed polygon; **(E)** inclusion of curvature dynamics $\left[\dot{\psi_{j_{1}}},\dot{\psi_{j_{2}}}\right]$ by overlaying previously generated image ***z***_*p*,*q*_ (*t* − *t*_*o*_) for a soft robotic fish profile with $\psi_{j_{1}}=0{}^{\circ}$ and $\psi_{j_{2}}=-20{}^{\circ}$ .

In order to overcome the above limitations and obtain the soft robotic fish’s continuous curvature incorporating the SCP actuators’ dynamics, this paper presents a novel state representation of the soft robot’s profile using grayscale images. These grayscale images are computationally generated such that they identically replicate the masked top view of the soft robotic fish, in order to speed up the training of the agent rather than depend on the visual processing/feedback from experiments on the robotic fish. First, as shown in Figure 5A, the three links of the fish are geometrically plotted using the joint angles $\left[\psi_{j_{1}},\psi_{j_{2}}\right]$ such that the vector of 2D coordinates $\left[X_{l},Y_{l}\right]\in R^{4\times 2}$ marks the vertices of the three links, where $X_{l}=\frac{1}{2}{\left[-|l_{2}|-2|l_{3}|\cos\psi_{j_{2}},-|l_{2}|,|l_{2}|,|l_{2}|-2|l_{1}|\cos\psi_{j_{1}}\right]}^{\text{T}}$ and $Y_{l}={\left[|l_{3}|\sin\psi_{j_{2}},0,0,|l_{1}|\sin\psi_{j_{1}}\right]}^{\text{T}}$ . Second, as shown in Figures 5A,B discretized set of 2D coordinates forming a perimetric offset around the three links are generated by applying a coordinate transformation function Λ(⋅) given by

$$\Lambda_{\text{x}}\left(X_{l}\right)=\rho \left[X_{l}+X_{d},\xi \cos\beta ,\left(X_{l}+X_{d}\right)J_{\left|\Lambda_{\text{x}}\right|}\right]+\frac{q}{2},$$ (19)

$$\Lambda_{\text{y}}\left(Y_{l}\right)=\rho \left[Y_{l}+Y_{d},\xi \sin\beta ,\left(Y_{l}-Y_{d}\right)J_{\left|\Lambda_{\text{x}}\right|}\right]+\frac{p}{2},$$ (20)

where *ρ* is the ratio between the maximum coordinates and required image size of dimensions *p* × *q*, $X_{d}=\left[-4\cos\psi_{j_{2}},0,0,\xi \cos\psi_{j_{1}}\right]$ , $Y_{d}=\left[4\sin\psi_{j_{2}},-1.5,-2,\xi \sin\psi_{j_{1}}\right]$ , *ξ* = 2.5, ***β*** = [−90°, −70°, *…*, 90°], and $J_{\left|\Lambda_{\text{x}}\right|}\in R^{\left|\Lambda_{\text{x}}|\times |\Lambda_{\text{x}}\right|}$ is a backward identity or standard involutory permutation matrix (Horn and Johnson, 2012). Next, the generated offset coordinates are interpolated and characterized by a cubic spline algorithm, which can be easily achieved using predefined functions in commercial simulation software such as interp1 in Matlab, thus forming a streamlined airfoil-like boundary of a fish as shown in Figure 5C. Finally, the interpolated coordinates form a polygon which is the Region of Interest (RoI) and can be converted to a binary image matrix $z_{p,q}\in Z^{p\times q}$ where ***z***_*p*,*q*(i,j)_ ∈ (0, 1) refers to the (i, j)^th^ entry of the image matrix, by applying a masking function such as poly2mask in Matlab. However, for further discretized transformations and grayscale image processing, the generated image domain is mapped to the R space such that ***z***_*p*,*q*_↦*f* (***z***_*p*,*q*_) and f:Z→R . The generated image now illustratively exhibits the curvature profile of the soft robotic fish as shown in Figure 5D. In order for the learning agent to acquire knowledge on the curvature dynamics also, the temporal information comprising the flapping angular velocities $\left[\dot{\psi_{j_{1}}},\dot{\psi_{j_{2}}}\right]$ is embedded onto the same image by overlaying the previous frame as shown in Figure 5E. For the purpose of brevity, if the entire image generation process at time *t* is mathematically denoted as Φ(***ψ*** (*t*)), then the overlayed image generated at time *t* is given by

$$z_{p,q}\left(t\right)={sat}_{0}^{1}\left(\right.\frac{1}{2}|4\Phi \left(\psi \left(t\right)\right)-\Phi \left(\psi \left(t-t_{o}\right)|\right),$$ (21)

where ${sat}_{0}^{1}\left(\cdot \right)$ denotes the saturation function limiting every pixel in the range (0, 1), and *t*_*o*_ is the time interval between two subsequent observations. The state observation input to the learning agent, thus becomes a concatenated structure of the image matrix, and a function of the system state and output reference vectors such that ***s*** = *f* (Φ(***ψ***), ***x***, ***y*** ^∗^).

#### 5.1.3 DDPG Learning-Based Controller Design

**FIGURE 6:**
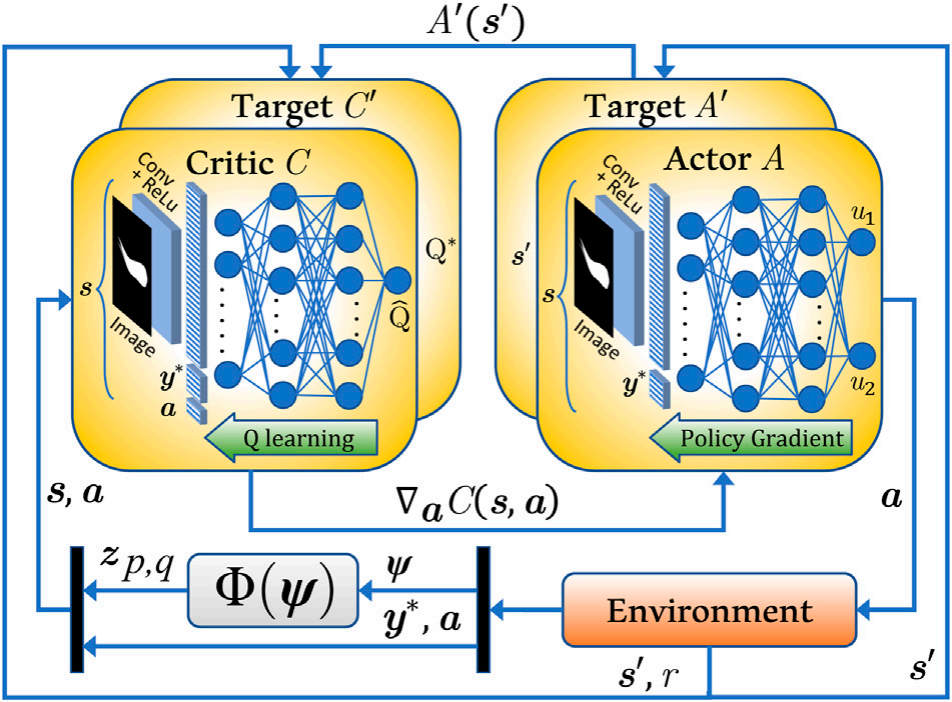
DDPG process chart incorporating image-based observations.

The DDPG algorithm (Lillicrap et al., 2015), as illustrated in Figure 6 and elucidated in Algorithm 1, primarily employs the use of a critic *C* and an actor *A* neural network. Due to the image-based observational input to the agent, the actor neural network is modeled as a combination of a convolutional neural network (CNN) and a DNN as shown in Figure 6. The algorithm inputs the grayscale image matrix ***z***_*p*,*q*_(*t*) to the CNN and performs a sequential convolution on the image with a kernel or filter of size *k*_*f*_ at a stride of length *k*_*l*_ to extract the features from the image. The convolved image goes through a pooling layer, fully flattened, concatenated with the rest of the state vector *f*(***x***, ***y*** ^∗^), and is then collectively fed to the actor DNN. Throughout the agent’s life span *t*_total_ which constitutes one training episode, the actor estimates the best action ***a*** at every time step *t*_***a***_ that can be carried out in a given state ***s*** as per its most recently trained policy *π*_*f*_, aka the representation of state-action mapping. An Ornstein-Uhlenbeck noise process of variance *σ* ^2^ is induced to the selected action to influence global exploration while training. The agent performs the chosen action by executing the soft robotic fish dynamics as described in Eqs 4–18 stepping through a time interval of *t*_***s***_ where *t*_***s***_ ≪ *t*_***a***_, followed by which the environment returns a new state ***s*** ′ and a reward *r*. These entities collectively establish a transition tuple *ɛ* = (***s***, ***a***, *r*, ***s*** ′) that is incrementally stored in a huge dataset known as the experience replay buffer **E**. At every action time *t*_***a***_, a mini-batch **E**_mb_ of *n*_mb_ transitions is randomly sampled from **E**, and its targets are determined from the Bellman equation (Lillicrap et al., 2015). A mean-squared error loss between the target values and its estimates are determined and back-propagated through the critic network *C*. The propagated gradients of the updated critic network are then used to reform the actor network. A recent target replica of the actor *A*′ and critic *C*′ DNNs are retained to chase a set of temporarily fixed targets, thus encouraging convergence of the algorithm. The overall training lasts for *N* episodes, with a terminal condition based on a reward averaged over a set of latest episodes.

Algorithm 1 Deep-Deterministic Policy Gradient Learning in Soft Robotic Fish

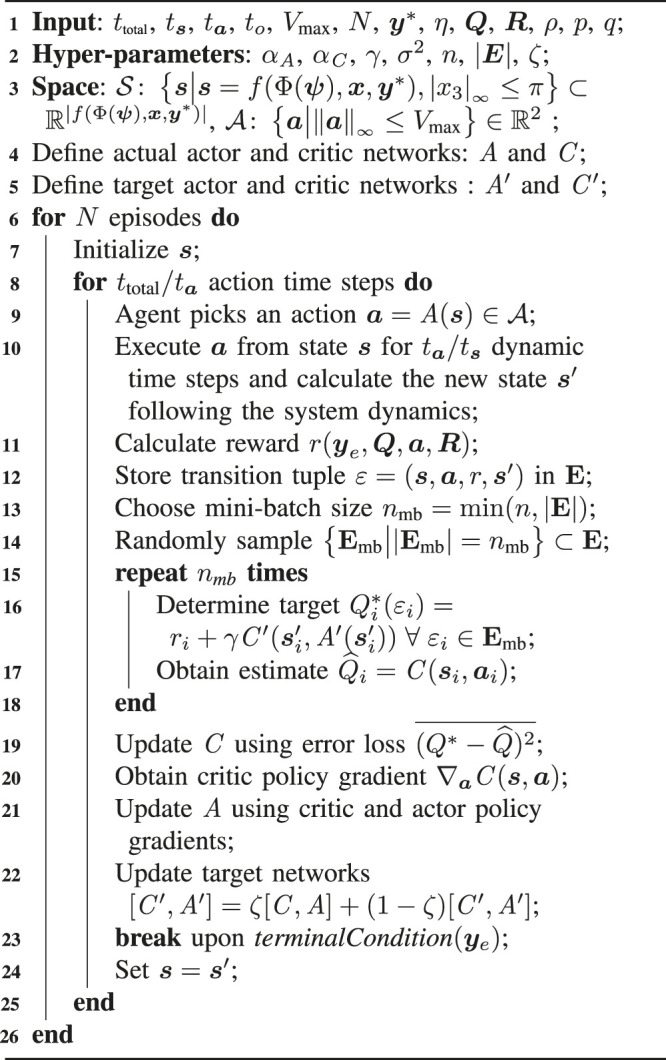

### 5.2 Reward Function

The shaping of the reward function plays an important role in training the agent. The high nonlinearity of the aforementioned modeled soft robotic fish, selects in this paper a reward *r* equipped with a linear quadratic regulator (LQR) cost function given by

$$r=-\eta \left(y_{e}^{\text{T}}Qy_{e}+u^{\text{T}}Ru\right),$$ (22)

where *η* is a scaling factor, ***y***_*e*_ = ***y*** ^∗^ − ***y*** is the tracking error of the system output, and ***Q*** and ***R*** are the weight matrices bringing in a trade-off between the system performances and control input efforts respectively.

### 5.3 Hyper-Parameters

Hyper-parameters play a significant role in the duration of training and accuracy of finding a global optimum and convergence. These parameters include the learning rate of the critic *α*_*C*_ and actor *α*_*A*_ networks such that *α*_*C*_, *α*_*A*_ ∈ (0, 1), whereby very small learning rates increase the chance of global exploration, hence decreasing the chances of reaching local optima. Several other parameters are the size of the experience buffer |**E**| which provides adequate sampling space, size of the sampled minibatch *n* which are generally chosen in powers of 2 to favor computational efficiency, reward discount factor *γ* which denotes the significance of the far rewards over the near rewards, variance of the noise process *σ* ^2^ to control the exploration factor, number of episodes for averaging of reward, and terminating criterion of the training pertaining to the averaged reward.

## 6 Simulation Results

This section presents the simulation results of two control tasks—yaw control and path following, to evaluate the performance of the proposed DDPG-based control of the soft robotic fish. The two control objectives serve as fundamentally decomposed control goals in high level control objectives such as path planning, schooling, shoaling, leader-following, etc. Table 1 shows the parameters applied in the simulations, which pertain to the environment, learning hyper-parameters, SCP muscles and fish dynamics. The thermo-electric and thermo-mechanical SCP muscle parameters follow (Rajendran and Zhang, 2017; Yip and Niemeyer, 2017; Rajendran and Zhang, 2018). While some of the training hyper-parameters adopt (Lillicrap et al., 2015), others are chosen by trial and error to expedite the convergence of the training by weighting the level of global exploration versus local exploitation. The fish dynamics parameters, however, are designed by envisioning the soft robotic fish and its expected planar motion comprising the hydrodynamic coefficients, and approximating the parameters of previously modeled robotic fish which exhibit similar motions (Marchese et al., 2014).

**TABLE 1:** Simulation parameters.

Definition	Symbol	Value (unit)
Soft robotic fish system design parameters		
Action time step	*t*_*a*_	0.5 s
Dynamics simulation time step	*t*_*s*_	0.01 s
Observation interval	*t*_*o*_	0.5 s
Duration of episode	*t*_total_	300 s
Maximum voltage to SCP	*V*_max_	25 V
Image observation parameters		
Dimension of image	*p*	128 pixels
Dimension of image	*q*	128 pixels
Image coverage ratio	*ρ*	5
Convolution kernel size	*k*_*f*_	8 × 8 pixels
Convolution stride length	*k*_*l*_	2 pixels
Training/Hyper-parameters		
Size of minibatch	*n*	128
Size of experience buffer	|**E**|	1,000,000
Learning rate of actor	*α*_*A*_	0.000 1
Learning rate of critic	*α*_*C*_	0.001
Target smooth factor	*ζ*	0.001
Actor noise variance	*σ* ^2^	0.8
Far/near reward discount factor	*γ*	0.99
Actor DNN hidden layer size	—	300 × 400
Critic DNN hidden layer size	—	300 × 400
Reward scaling factor	*η*	1.212 9 × 10^–7^
Fish dynamics parameters		
Length of each link	|*l*|	5 cm
Mass of the robotic fish	*M*_*f*_	10 g
Added mass along x axis	*M*_x_	0.85 g
Added mass along y axis	*M*_y_	1.25 g
Mass moment of inertia	*J*_*z*_	0.003 5 g.cm^2^
Coefficient of drag force	*K*_*D*_	0.5 g.cm^2^
Coefficient of drag along *α*	$K_{D_{\alpha }}$	0.000 7 g.cm
Coefficient of lift force	*K*_*L*_	2.17 g.cm^2^
Coefficient of thrust force $F_{T_{1}}$	$K_{j_{1}}$	0.004 g.cm^2^
Coefficient of thrust force $F_{T_{2}}$	$K_{j_{2}}$	0.05 g.cm^2^
Damping coefficient	*K*_*M*_	0.001 05 g.cm^2^
Moment coefficient of *j*_1_	$K_{M_{1}}$	0.25 cm
Moment coefficient of *j*_2_	$K_{M_{2}}$	0.25 cm
SCP actuator dynamics parameters		
Original length of muscle *m*_*i*_	*L*_0_	10 cm
Mass of SCP actuator	*M*_*m*_	0.05 g
Electrical resistance	*R*_*m*_	8 Ω
Thermal mass	*C*_th_	0.5 W.s/°C
Absolute thermal conductivity	*λ*	0.85 W/°C
Mean stiffness	*k*_*m*_	1.65 N/m
Damping coefficient	*b*_*m*_	1.1 N.s/m
Thermal constant	*c*_*m*_	0.03 N/°C
Ambient temperature	*T*_0_	25°C

The system design parameters are selected considering the reasonable SCP dynamics in conjunction with the fish flapping tail frequency, thus having an action time step of *t*_***a***_ = 0.5 s. The image observation parameters are chosen based on the performance of the CNN and foreseeing the computational processing power of a hardware computer vision/image processor such as OpenMV, Pixy, and Raspberry Pi Cameras to generate image-based observations. Regardless of the camera used in the experiments, they all support a minimum capture rate of 60 frames per second (FPS), thus giving a wide window of time to determine the next action ***a*** given an observation ***s***, and therefore, deeming the proposed visual learning-based control algorithm realizable due to the considerable sampling time *t*_*o*_ = *t*_***a***_.

### 6.1 Yaw Control

The yaw control objective of the soft robotic fish aims at orienting the robot at a desired angle such that *θ* ^∗^ ∈ [−*π*, *π*]. As this requires the agent to obtain the knowledge of both the current angle *θ* and desired angle *θ* ^∗^ as part of its observation ***s***, the learning is subtly modified to reduce the dimension of the observation ***s*** for quicker convergence. Consequently, the observation comprises of the difference between the current and desired angles such that the agent’s target remains *θ* ^∗^ = 0 at all times, whereas the agent itself is randomly initialized to θ∼U[−π,π] following a uniform distribution at the beginning of its lifespan. The state observation thus becomes $s=\left\{\Phi \left(\psi_{j_{1}},\dot{\psi_{j_{1}}},\psi_{j_{2}},\dot{\psi_{j_{2}}}\right),y^{*}\right\}$ , which includes the image containing the curvature dynamics and the system output target vector such that $y^{*}={\left[\omega_{\text{z}}^{*},v_{\text{total}}^{*},\alpha^{*}\right]}^{\text{T}}\in R^{3}$ . As for the yaw control task, we select ***y*** ^∗^ = (0, 2, 0) in this paper. The LQR-based reward weights are set to ***Q*** = diag (2, 0.05, 2000, 0.01) and ***R*** = diag (0.001, 0, 0.001, 0). These weights are manually tuned such that the yaw angle and total velocity are weighted more than the rest of the outputs. The rest of the system states and dynamics of the soft robotic fish are initially reset to zero at the start of every episode. A training episode is conditionally terminated betimes upon satisfying *terminalCondition* $\left(y_{e}\right)=\left(\left(\theta^{*}-\theta \right)\leq \tilde{\theta }\right)\vee \left(v_{\text{total}}\geq v_{\text{total}}^{*}\right)$ , where $\tilde{\theta }$ is the acceptable threshold of angular orientation and its bounds are set to ±10°. The agent was trained for 5,000 episodes with each episode lasting for 300 s, and embarked convergence just after 250 episodes while encouraging local exploitation throughout the rest of the episodes.

**FIGURE 7:**
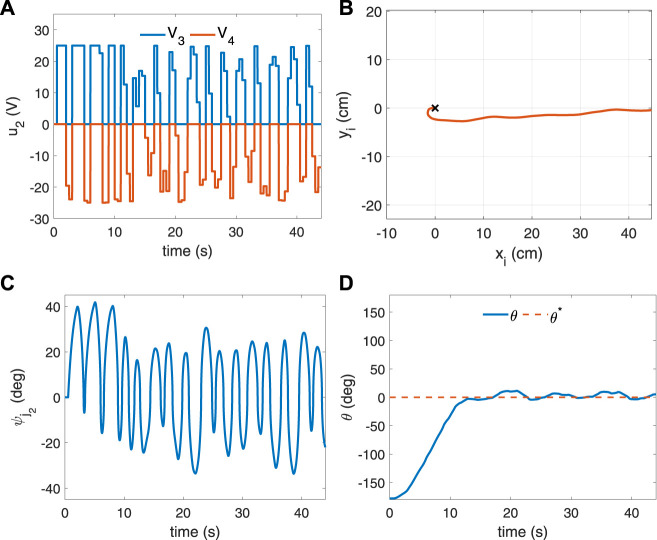
Simulated result of yaw control of the robotic fish initialized at the origin with pose (x_i_, y_i_, *θ*) = (0, 0,−178°) and desired orientation *θ* ^∗^ = 0°. **(A)** Control input *u*_2_ representing the voltages of the SCP muscles *m*_3_, *m*_4_; **(B)** the trajectory of the robotic fish turning from −178° to 0°; **(C)** the tail flap angle $\psi_{j_{2}}$ ; **(D)** the yaw angle of the fish *θ*.

The trained agent is then simulated to control the soft robotic fish, initialized at (x_i_, y_i_, *θ*) = (0, 0, −178°), to achieve a desired orientation of *θ* ^∗^ = 0°. The control input *u*_2_ generated by the actor network is shown in Figure 7A and the corresponding change in the tail angle $\psi_{j_{2}}$ due to the SCP muscles contractions is plotted in Figure 7C. The entire trajectory of the soft robotic fish for the given control input is shown in Figure 7B with the current and desired orientations shown in Figure 7D. The simulated result of yaw control of the soft robotic fish is also animated in Video 1 which is included in the Supplementary Materials. As it can be observed from these results, the agent exhibits a learned swimming profile to orient the fish at 0° and achieves convergence by reaching the target angle within 13 s, *via* coordinated actuation of the SCP muscles *m*_3_ and *m*_4_.

**FIGURE 8:**
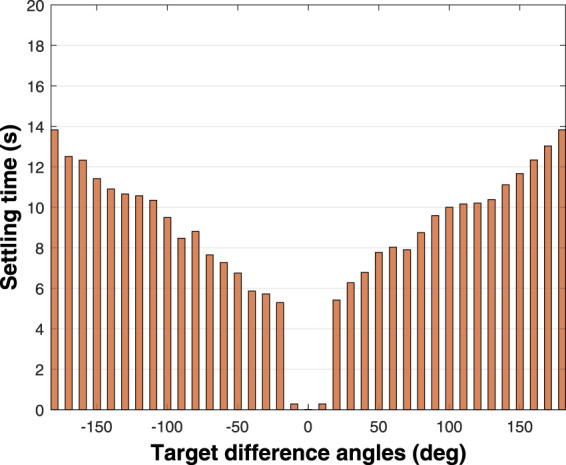
Simulated result of the settling times in yaw control of the soft robotic fish initially oriented at zero degrees and targeted to swim at every angle spaced by 10 degrees in the range (−180°, 180°).

The overall performance of the trained agent is evaluated by simulating the soft robotic fish for 60 s, initialized at 10 degree intervals in the range (−180°, 180°), with its desired angle set to zero at all times. Two performance factors are taken into consideration pertaining to the yaw angle regulation: 1) settling time, and 2) steady state error. The settling times of all these simulated periods are collated by obtaining the time instants when *terminalCondition* is satisfied, and the resulting plot is illustrated in Figure 8. Evidently, as shown in the figure, we see that it only takes 20 s for the soft robotic fish to rotate 180 degrees based on the dynamics described in Eqs 4–18. Additionally, as the difference between the current and desired orientation angle increases, the settling time also increases. We also find that the outcome slightly favors negative values of desired angles over the positive values, thus appearing asymmetrically, which can be attributed to algorithm’s randomness such as initialization of the actor and critic neural networks’ weights before the training, the shift in algorithm’s Q-value during training, and convergence of the training based on the samples selected in the experience replay buffer. In order to balance this predicament, prolonged training of the agent is encouraged to refine the convergence with minimal shift in the actor NN’s weights.

**FIGURE 9:**
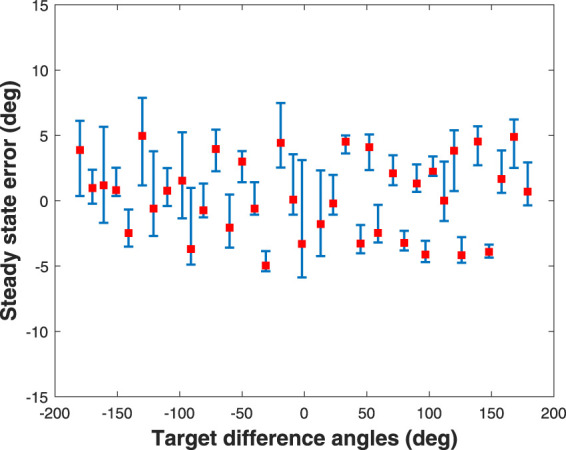
Simulated result of the steady state errors in yaw control of the soft robotic fish initially oriented at zero degrees and targeted to swim at every angle spaced by 10 degrees in the range (−180°, 180°), where error bars represent the steady state boundaries caused due to the flapping oscillations.

The outcome of the evaluation in terms of the steady state error in the angular orientation is shown in Figure 9, where the steady state errors of the soft robotic fish agent at different target angles spaced at 10 degree intervals in the range (−180°, 180°) are collated and displayed using red squares. The error bars corresponding to each target angle represent the steady state boundaries caused due to the flapping oscillations. As the minimization of the angular velocity or swinging motion is essential to alleviate the effect of the hydrodynamic drag force which reduces propulsive efficiency (Liu et al., 2008; Farideddin Masoomi et al., 2015), we see that throughout the range of the soft robotic fish’s target angles, the agent has learned to maintain a steady state error within ±5 degrees satisfying $|\;\tilde{\theta }\;|\;\leq \;5{}^{\circ}$ , thus proving the agent’s robustness. The difference in the error bounds at different target angles can again be attributed to the stochasticity in the initialization of the neural networks and the soft robotic fish, and can be mitigated *via* prolonged training of the agent.

### 6.2 Path Following

**FIGURE 10:**
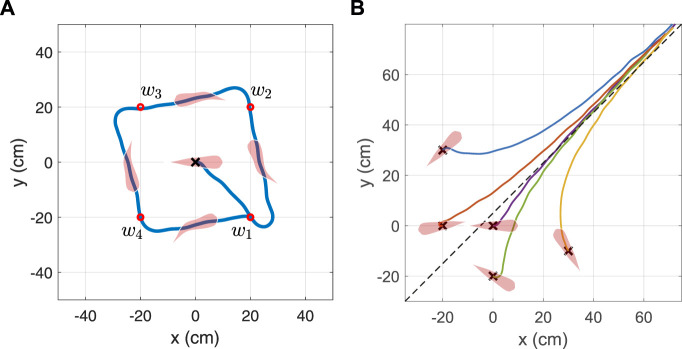
Simulated result of the robotic fish following a path defined by **(A)** a cyclic set of four waypoints and **(B)** a line defined by the equation −x_i_ + y_i_ = 5.

As the trained agent is capable of successfully controlling the orientation of the soft robotic fish, this section demonstrates the agent’s ability to continuously follow a predefined path. Hence, the agent is strenuously tested by simulating the robotic fish to follow a set of planar waypoints closely constrained and proportional to its body length (BL) in order to observe the maneuvering range. In the first test, four waypoints are generated and arranged equidistantly to the origin and subsequent preceding and succeeding waypoints. The robotic fish is initialized at the origin with the pose (x_i_, y_i_, *θ*) = (0, 0, 0°), and set to follow the waypoints numbered (*w*_1_, *w*_2_, *w*_3_, *w*_4_) in a cyclic manner. The target angle is determined at every action time step *t*_***a***_ given by $\theta^{*}=\tan^{-1}\left(\frac{y_{w_{n}}-y}{x_{w_{n}}-x}\right)$ , where $\left(x_{w_{n}},y_{w_{n}}\right)$ mark the 2D coordinates of the current target waypoint *w*_*n*_ in the inertial frame $F_{\text{i}}$ satisfying *n* ∈ (1, 2, 3, 4). Once the fish reaches within 1 cm radius of its current target waypoint *w*_*n*_ satisfying $\sqrt{{\left(x_{w_{n}}-x_{\text{i}}\right)}^{2}+{\left(y_{w_{n}}-y_{\text{i}}\right)}^{2}}<1$ , a new waypoint *w*_*n*+1_ is assigned as the next target to the agent. The simulated result, as illustrated in Figure 10A and animated in Video 2 of Supplementary Materials, shows the agent reaching all the waypoints where each segment is constrained to a little over 2BL.

Following this, a second test is performed to test the agent to follow a line defined by the parametric equation *g*_1_x_i_ + *g*_2_y_i_ + *g*_3_ = 0, when initializing the soft robotic fish to different poses (x, y, *θ*). At every action time step *t*_***a***_, the cross-track error (CTE) which is defined as the normal distance between the center of the fish and the target line, is computed by

$$\text{CTE}=\frac{\left[g_{1},g_{2},g_{3}\right]\cdot \left[x,y,1\right]}{\sqrt{g_{1}^{2}+g_{2}^{2}}},$$ (23)

which leads to our design of the target orientation of the fish $\theta^{*}=\tan^{-1}\left(\frac{-g_{1}}{g_{2}}\right)-2{sat}_{0}^{10}\left(\text{CTE}\right)$ . The result of this outcome, as shown in Figure 10B, demonstrates the agent starting in different poses, eventually converging to the target line minimizing the CTE.

## 7 Conclusion

This paper proposed a novel design of a soft robotic fish actuated by antagonistically arranged SCP artificial muscles, which takes advantage of the quicker heat dissipation in SCPs when submerged in water, thus leading to faster actuation. The soft robotic fish was modeled from its geometrical and dynamical perspectives to realize a two-dimensional swimming motion by incorporating hydrodynamic forces and moments. The paper also presented a learning-based controller design, which perceives the curvature dynamics and soft profile of the fish *via* image-based state observations. We conjecture that this type of visual learning-based controller design can be generalized and ubiquitously used in training/inference of agents to self-learn locomotion in soft robots that are limited with volumetric constraints and pose challenges in embedding complex curvature-sensing electronics. Not only this sensing approach leads to more flexible and less expensive soft robots, but also contributes towards decrease in the production time. Additionally, the derived model and learning-based controller were simulated to evaluate the agent’s performance and validate its effectiveness with respect to two control objectives i.e., regulating the robot’s yaw angle and following a predefined path.

The future scope of this paper branches out to several directions such as optimal design of SCP-actuated soft robots and researching online reinforcement learning-based controllers. Significantly, the visual learning-based controller design could pave a path to embark on a new research direction towards visual imitative learning in soft robots from real biological lifeforms, thus not only mimicking the anatomical functions, but also mimicking the cognitive phases in locomotion and social behavior. Nevertheless, our future research work primarily includes culminating the development of the experimental platform to test the SCP-driven soft robotic fish by addressing some current impediments such as buoyancy control and mobile power supply, followed by validating the proposed visual learning-based controller design in real-time. Concurrently, we also plan to investigate the design, outcome and performance of a fully image-based state feedback controller to simplify the learning approach by reducing the number of required embedded positional sensors, aiming to expand its applications to a wider variety of soft robots.

## Data Availability

The raw data supporting the conclusion of this article will be made available by the authors, without undue reservation.
